# Root architecture and visualization model of cotton group with different planting spacing under local irrigation

**DOI:** 10.3389/fpls.2023.1080234

**Published:** 2023-04-21

**Authors:** Chao Gao, Mingsi Li, Dongwei Li

**Affiliations:** ^1^ College of Agricultural Science and Engineering, Hohai University, Nanjing, China; ^2^ College of Water Conservancy and Architecture Engineering, Shihezi University, Shihezi, China; ^3^ Farmland Irrigation Research Institute, Chinese Academy of Agricultural Sciences, Xinxiang, China

**Keywords:** planting spacing of cotton group, root density, soil water suction, root system architecture, cellular automata

## Abstract

Planting spacing plays a key role in the root system architecture of the cotton group under local irrigation. This study used the Cellular Automata (CA) theory to establish a root visualization model for the cotton group at two different planting spacing (30 and 15 cm) within a leaching-pond. At a planting spacing of 30 cm, the lateral roots grew almost horizontally toward the irrigation point, and a logarithmic relationship was observed between root length density and soil water suction. However, at a planting spacing of 15 cm, the lateral roots exhibited overlapping growth and mainly competed for resources, and a power function relationship was observed between root length density and soil water suction. The main parameters of the visualization model for each treatment were essentially consistent with the experimental observations, with respective simulation errors were 6.03 and 15.04%. The findings suggest that the correlation between root length density and soil water suction in the cotton plants is a crucial driving force for the model, leading to a more accurate replication of the root structure development pathway. In conclusion, the root system exhibits a certain degree of self-similarity, which extends into the soil.

## 1 Introduction

The root is an important organ of the plant for obtaining water and nutrients from the soil and has the function of anchoring the plant. The efficiency of water and fertilizer absorption in plants is significantly influenced by the spatial distribution of their root system (Fernández and Simmonds, 2006; Liang et al., 2007; Yan et al., 2007). Observing the root growth process of field crops is challenging, which can hinder effective irrigation and fertilization of the root zones. Uneven soil moisture resulting from localized irrigation can lead to an asymmetric distribution of root architecture (Ning et al., 2015; Zanetti et al., 2015; Khan et al., 2016; Meshram and Mahajan, 2017), which adversely impact the plant’s stability and the water uptake efficiency of its root system. The uniformity of the soil wetting zone affects the distribution of roots, agronomic traits (such as plant height, leaf area, harvest index, and total biomass), as well as the accumulation of total photosynthetic products and ultimately the yield of the crop (Liu et al., 2006; Sun et al., 2014). Therefore, a precise technical designing of a localized irrigation and fertilization system can facilitate control over the morphological development of root systems (Lynch and Brown, 2012; Smith and De Smet, 2012; Wen et al., 2015).

The competition among plants plays a vital role in shaping the structure and dynamics of a community, especially with regard to the availability of nutrients and water in the soil and assimilation of such resources by competing root systems (Hortal et al., 2017). Roots are dynamic, tip-growing structures located at the base of a plant that facilitate the absorption of water and nutrients from the soil (Thomas, 2017). The soil environment impacts plant competition for soil resources and changes root growth (Larios and Suding, 2015), presenting gravitropism and hydrotropism, respectively (Su et al., 2017). Gravitropism is an important response of plant growth to the soil environment, which drives the roots downward, allowing the roots to reach the soil for its primary function (Chen et al., 1999; Strohm et al., 2013). Hydrotropism is the growth response of a plant in which the growth direction is determined by a stimulus or a gradient in the concentration water, showing a growth regulated by the roots toward the humid zones (Miyazawa et al., 2011). Hydrotropism, among other root tropisms, can be considered as a direct mechanism for drought avoidance.

Advances in computing power have helped to model explicit root architecture (Cao et al., 2008; Li et al., 2011). A three-dimensional (3D) morphological model of rice roots based on Visual C++ 6.0 was constructed by observing and analyzing the morphological characteristics of roots treated with various varieties, water and nitrogen. The model extracted morphological parameters from the rice roots (Xu et al., 2010). This research not only enabled the 3D visualization of the growth process of rice roots but also established the foundations for developing a comprehensive system for visualizing rice root growth. Kalbacher et al. (2011) developed a coupled root–soil growth model based on the Richards equation. These findings formed the basis for a multi-root competitive growth model within a spatial network. Tan et al. (2011) used the platform of OpenGL and realistic visualization technologies to integrate the parameters and topology of a root morphology model to construct a 3D display model of the wheat root axis, thus enabling the 3D visualization of the wheat root system. Dupuy et al. (2010) developed a growth and function root growth model based on the dynamic relationship between the root density distribution and individual root development parameters.

The SimRoot model can be used to simulate the root architecture of the 40-day crop for soybean (tap root system) and maize (fibrous root system) (Postma and Lynch, 2010). The Lindenmayer System (L-system) in fractal theory is widely used for establishing the visual models of plant root architecture. Leitner et al. (2010) developed a plant root growth model based on the L-system using the modular approach and defining the growth rules, and the results can be applied to different soil types. Zhong et al. (2008) established a 3D simulation system that employs L-system differential system theory combined with infographics to visualize the growth of soybean root growth. This approach resulted in an intuitive and continuous graphical representation of soybean root growth. However, current research on root modeling has primarily concentrated on individual plant root system, with the majority of studies focusing on fibrous root systems. These investigations have explored root growth, morphological structure, and tropism using simulation studies. Nonetheless, there is a dearth of literature on the simulation of root structure in crop groups, especially when it comes to applying Cellular Automata (CA) theory. L-system is a computerized reduction of root architecture based on fractal theory and parameters are known independent of its extrinsic environmental conditions. CA is an effective tool for modeling root growth under varying soil environmental conditions, such as stress and drought. This method characterizes the spatial distribution of water stress, which guides the root’s tropism as it moves through the soil environment. In fact, root growth is predominantly driven by hydrotropism, which responds to the water status of the soil environment. When evaluating root growth in plant group located in the soil wetting zones, it is essential to consider the impact of root competition for water and fertilizer resources, as this competition is primarily driven by hydrotropism. Root growth and root water absorption are tightly interconnected processes. Roots tend to grow in areas of high moisture content or humidity. Therefore, we hypothesized that the root system of the cotton group is predominantly characterized by tropic growth. To describe the distribution characteristics of the root system of the cotton group in the soil wetting zones, we present a conceptual model of root architecture using the CA theory.

The objectives of this study were (1) to investigate the relationship between the root length density of cotton group and soil water suction, (2) to establish the root system architecture model by CA theory, and (3) to reproduce the development route of the cotton’s group root system architecture.

## 2 Materials and methods

### 2.1 Experimental design

**Figure 1:**
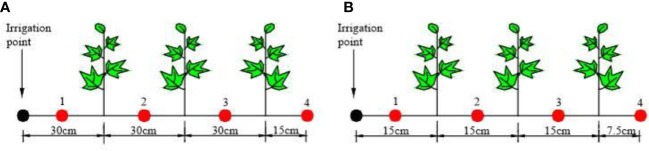
Cotton planting spacing and sampling point setting. **(A)** 30-cm planting spacing. **(B)** 15-cm planting spacing.

The experiment was carried out from April to September 2015 in the Key Laboratory of Modern Water-saving Irrigation of Shihezi University (86˚03’27”E, 44˚18’25”N; Altitude 451 m). The cotton variety was “Xinluzao 23”. In the growing period of the cotton (May–August), the average rainfall was 15–30 mm. The annual rainfall was 180–270 mm and the annual evaporation was 1105.4 mm. The dimension of the leaching-pond was (length × width × depth, cm × cm × cm) 110 × 80 × 60 (**Supplementary Figure S1**). The experimental treatment design involved setting two different cotton planting spacing treatment: one with a spacing 30 cm and another with a spacing of 15 cm (**Figure 1**). Fifteen repetitions per treatment were used to the three growth stages of cotton (Bud stage, Flowering stage, and Boll stage, five repetitions per stage). Additionally, three repetitions are used to slice root and two repetitions are used to get the root architecture), resulting in a total of 30 leaching-ponds (2 × 15). There are three cotton plant in each of leaching-pond. To prevent groundwater interference, the bottom and surrounding areas of the leaching-pond were fully covered with plastic film. In order to form different soil wetting patterns, each treatment was irrigated from only one side of the leaching-pond at any given time (**Figure 1**, the irrigation point is fixed, relying entirely on matric potential to transport the water horizontally).

The experiment focused on observing and measuring three stages of cotton development that are known to have contrasting root development, Bud stage (June 24–July 11), Flowering stage (July 12–31), and Boll stage (August 1–18). The root system morphology is basically established at the Flowering stage. The irrigation and fertilization scheme for each leaching-pond in the experiment was the same. Fertilizer was poured in the water. The total amount of fertilization in each leaching-pond throughout the growing stage was Nitrogen fertilizer 119.6 g, with a nitrogen content of 46.4%, Phosphate fertilizer (ammonium potassium phosphate) 74.0 g, with a P_2_O_5_ content of 51.5%. The irrigation and fertilization scheme is shown in **Table 1**. To facilitate monitoring of the soil wetting area during irrigation, the soil surface was left uncovered with plastic film. Rain protection measures were taken for the cotton during the rainfall process to ensure effective water treatment.

**Table 1:** Irrigation and fertilization scheme.

Growth stage	Seedling stage	Bud stage	Flowering stage	Boll stage	Boll-opening stage
Irrigating water quota (L)	8	10	13	13	14
Irrigation amount (L)	16	40	52	52	28
Irrigation cycle (d)	5	4	4	3	8
Nitrogen fertilizer (g)	13.2	26.4	32.0	32.0	16.0
Phosphate fertilizer (g)	8	16	20	20	10

### 2.2 Experimental method

#### 2.2.1 Soil

Although three cotton plants were present within the soil wetting zone, there was a noticeable difference in soil moisture levels between the areas near and far from the irrigation point in the root zone. According to the USDA textural soil classification (Soil Survey Staff, 2011), the percentage content of clay particles (particle size< 0.002 mm), silt particles (particle size 0.002–0.05 mm) and sand particles (particle size 0.05–0.25 mm) of the experimental soil were 13.36, 37.91, and 48.73%, respectively. So the soil texture in the experiment was medium loam. The soil bulk density was 1.589 g/cm^3^, porosity was 42.8%, and field capacity was 26.73% (volume water content). The soil moisture characteristic curve was measured with the 1500 F1 pressure film instrument produced by the American Soil Moisture Equipment Corp. (SEC) company. Given that the soil used for each experimental treatment was collected from the same field, three replicates were performed for each leaching-pond, with a total of 30 leaching-ponds being installed. van Genucheten (1980) model was used to fit the soil water characteristic curve. The relationship between soil water suction and soil volumetric water content was fitted by the exponential function distribution equation. The equation was as follows:


(1) $$\text{θ}={\text{θ}}_{r}+\left({\text{θ}}_{s}-{\text{θ}}_{r}\right){\left[1+{\left(\alpha \cdot S\right)}^{n}\right]}^{-\left(1-\frac{1}{n}\right)}$$

Where, θ is soil water content; ${\text{θ}}_{\text{r}}$ is soil residual water content; ${\text{θ}}_{s}$ is soil saturated water content; α, n are independent fitting parameters; S is the soil water suction, $S=\frac{\text{θ}-{\text{θ}}_{r}}{{\text{θ}}_{s}-{\text{θ}}_{r}}$ . Fitting parameters from experimental data are shown in **Table 2**.

**Table 2:** The parameters of equation (1).

θ*_r_*	θ*_s_*	α	*n*	*R*
0.219	0.447	7253.248	1.313	0.995

The soil was sieved and the leaching-pond was filled with the sieved soil at the end of April 2015. Seeds were sown on May 9, seedlings were fixed on June 10, and then irrigation began.

#### 2.2.2 Root

The vertical distribution of soil moisture was determined by the oven drying method. To obtain the vertical distributions of soil moisture, soil sample was collected at 10 cm intervals up to a depth of 60 cm, using a custom-made soil core with a diameter of 1 cm to minimize disruption of root growth within the soil. The horizontal sampling points were at the irrigation point and the middle position of the cotton plant, respectively (the sampling points 1, 2, 3, and 4 are shown in **Figure 1**).

**Figure 2:**
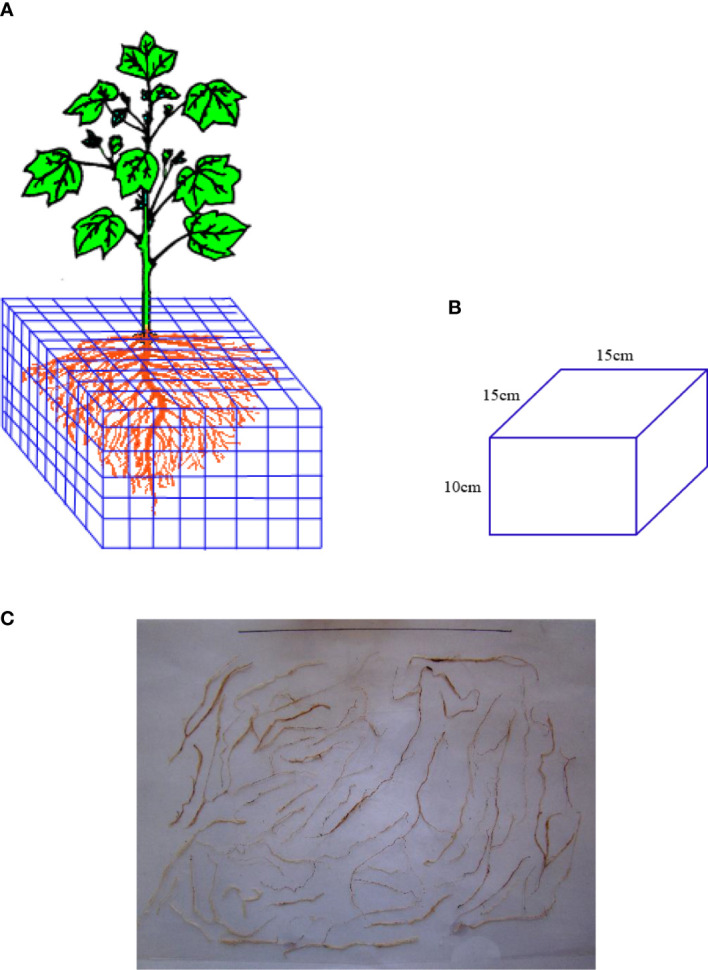
Methods for root imagens analyses. **(A)** Schematic diagram of sampling and sectioning. **(B)** Soil sample after slicing. **(C)** Root samples with scaled paper for taking pictures.

The grid bidirectional soil sectioning method and the excavation method were used to obtain root samples. Bidirectional grid soil sectioning method was used to obtain root samples for root density distribution analysis. The total root was divided by bidirectional slicing method (**Figure 2A**); the root samples were collected horizontally from plant within a width of 15 cm and vertically at a depth of 10 cm until the edge of the leaching-pond was reached, with a volume of 15 cm × 15 cm × 10 cm being collected for each sample (**Figure 2B**). The root samples were rinsed and then placed on scaled paper to take pictures after collecting and cleaning them (**Figure 2C**). The camera details are Nikon COOLPIX, NIKKOR, 3X OPTICAL ZOOM, 6.2–18.6 mm, 1: 2.8–5.2 Long Lens, 3x Optical Zoom, 1600 dpi, and ISO 125-1600), followed by vectorization of the root images using R2V software. Finally, the vector result files were processed with Microsoft Office Access 2016, and the root length was calculated. The root lengths in each section were added to get the total root length. The total root length divided by the corresponding soil volume is the root length density. All the root samples were dried in an oven at 65°C at a constant mass. The root dry weight was weighed with an electronic balance of 0.001 g. Then, the root weight density was measured.

The process of root architecture of whole plant extraction is as follows: selecting a cotton plant for sampling involves using an iron shovel to dig around the side roots at the bottom of the cotton plant, clearing the soil of debris and large particles, and gently cutting the cotton root system to avoid damage. Once the whole root system is exposed, it should be carefully removed from the soil while cleaning the surrounding area. Any damaged or injured roots should be removed with scissors, and only healthy and intact roots should be retained. Finally, the entire cotton root system should be sent to the laboratory for analysis. The length of both the main root and lateral roots was measured using a tape measure, while the diameter of these roots was measured with a vernier caliper that has an accuracy of 0.02 mm. The angle between the lateral root and the main root in the depth direction was measured using a protractor.

#### 2.2.3 Root length density and root weight density

The root length density of cotton group at 30- and 15-cm planting spacing was measured, respectively. As root weight was measured for all root samples from bidirectional grid soil sectioning method of all soil samples in the leaching-ponds experiment, while root length was only measured with three replicates per treatment, this study calculated the root length density by fitting the root length density and root weight density to obtain that for all samples. The root weight density was regression fitted to obtain the relationship between the root weight density and the root length density. Given the larger diameter of root axis, the corresponding relationship between root length density and root weight density at the root axis location was different from that at other locations. The regression fitting relationship was as follows.

Root axis location:


(2) RLD=831.37×ln(RWD)−1685.51    (R= 0.970)

Other locations:


(3) $$RLD=83.12\times RWD^{0.71}\;\left(R=\;0.860\right)$$

Where, *RLD* is the root length density, m/m^3^; *RWD* is the root weight density, g/m^3^.

#### 2.2.4 Root length density and soil water suction

The relationship between root length density and soil water suction was determined based on the principle of root tropism competition growth. Among them, the relationship between root length density and soil water suction was

30-cm planting spacing:


(4) $$R_{L}\left(S\right)=a\times \ln\left(\text{S}\right)+b$$

15-cm planting spacing:


(5) $$R_{L}\left(S\right)=c\times e^{d\times S}$$

Where $R_{L}\left(S\right)$ is the root length density, m/m^3^; *S* is soil water suction; MPa, *a*, *b*, *c*, and *d* are fitting parameters.

### 2.3 Model

**Figure 3:**
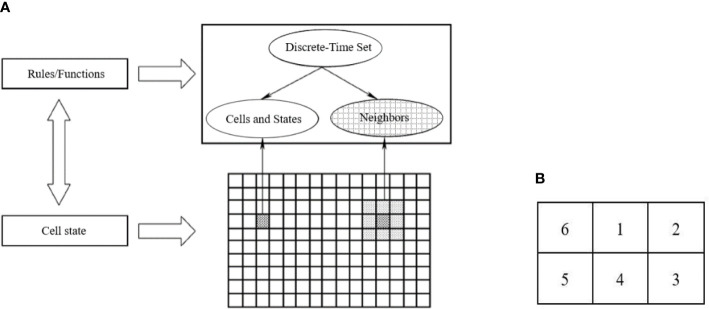
Cellular Automata model process. **(A)** The Composition diagram element of Cellular Automata. **(B)** Schematic diagram of evolution rules of Moore neighboring regions.

The CA model divides the space of motion into multiple cells using a specific grid form. This grid is well suited for dynamically simulating the spatiotemporal evolutionary process of complex systems. CA is a simulation method that uses local rules and connections, with each point on the grid representing a cell with a finite state. The rules of variation are applied to each cell and simultaneous. A typical rule of change depends on the status of the cell and its neighbors (4 or 8) (**Figure 3A**). The main features of a tuple automaton are as follows: (1) the space is discrete, (2) the time is discrete, (3) the states take values that are discrete, and (4) the rules of evolutionary operations are local. The programming code should be as simple as possible to minimize the number of operations.

#### 2.3.1 Basic theory of CA model

The CA model is composed of four key components: Cells, States, Neighbors, and Rule. Here, the soil is a cell in a cellular space. At any given time, a cell can only have one state, which is a finite state of the soil. Neighbors are defined as the cellular sets surrounding a cell, and they impact the cell’s state in the next moment. The Rule refers to the root growth dynamics within the soil grid. By following the same action rules and synchronous updating, every cell in the regular grid takes on a finite, discrete state. Through simple interactions based on local rules, a large number of cells form a dynamic evolutionary system. The CA model can be described in the following equation:


(6) $$CS_{t+1}=f\left(CS_{t},\;N\right)$$

Where *CS* is a finite set that it is individual cell state, *N* is the neighbor of the cells, *t* is the time, and *f* is the cellular move from the initial state to the next state under the local transformation rules, that it is section 2.3.2.

#### 2.3.2 CA model of the root growth of cotton group

The root growth model for the cotton group was modeled separately at different growth stages in this research. As a result, time was not considered when designing the CA model for the root growth process. The relationship between root length density and soil water suction was determined by fitting experimental data using Equation (4, 5), which was then used as the evolutionary rule in the CA model. In this paper, the CA model for simulating the dynamic changes in root growth can be defined as follows:


(7) CA=<Cells, States, Neighbors, Rule>

(1) Cells were the cell of cotton root growth, denoted as Cell (*x*, *y*) (where x is for horizontal and *y* is for vertical), representing 2D CA. Since root growth was irreversible, the determination of the cellular state in the CA model was also irreversible. The 2D space for simulating the root architecture of cotton group plants was as follows: horizontal direction is 110 cm (length of the leaching-pond), vertical direction is 60 cm (depth of the leaching-pond), the unit was determined as cm, and the cell size was 0.5 cm × 0.5 cm.
(2) States was the cell state. The classic model of CA was defined on a 2D grid (length × depth: 110 cm × 60 cm), and the state was represented by a matrix of 1 and 0. Where, 1 means there is a root, 0 means there is no root. The growth process of the root system in the CA model involved a change in the state value of the cellular unit from 0 to 1.
(3) Neighbors were the cell neighboring regions, which was the domain of Rules. The neighboring regions of the Cell (*x*, *y*) was denoted as N [Cell (*x*, y)]. The CA neighbors were all Moore neighboring regions (**Figure 3A**). That is, the 2D grid of the cotton root growth space in CA model assumed that cell 0 was the initial position of the cell, 1 was the start of growth, the root system grows in the direction of cells 2, 3, 4, 5, and 6 on the vertical plane (**Figure 3B**).
(4) Rule was the evolution rules of the CA model for the root growth of the cotton group determined the growth route of the root system. Each rule determined whether a cell would be a 0 or a 1 in the next generation, depending on the pattern. These rules are similar to totalistic CA, but they use continuous functions for both the rule and states, resulting in continuous states. The status of a location is represented by a finite number of real numbers. Using certain CA, it is possible to yield diffusion in liquid patterns.

According to the principle of hydrotropism for crop root growth, a relationship between the root length density of cotton group and soil water suction was formulated as the evolutionary rule for a CA model. Initially, a matrix representing the starting position of the roots (state 0) was determined by simulating the root growth process on a 2D soil space using CA theory. The rule governing the growth process was that the roots seek out moisture to facilitate growth (moving from state 0 to state 1). Finally, the root growth route for the cotton group was determined in the soil wetting areas (**Figure 3B**).

### 2.4 Statistical analysis

The data of soil and cotton plant were analyzed by using SPSS 22 and Excel 2016. The graphic representations of the data were produced by using Origin 2022b, and the model was implemented with MATLAB R2016a.

## 3 Results

### 3.1 Distribution of soil water suction

**Figure 4:**
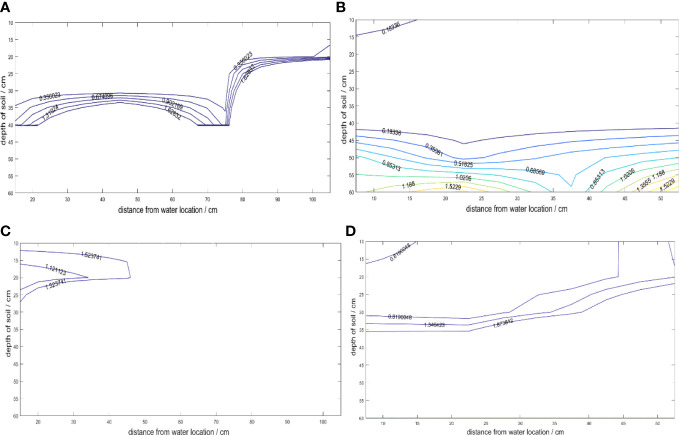
Contour map of soil water suction (MPa). **(A)** Flowering and Boll stage 12h after irrigation in 30-cm planting spacing. **(B)** Flowering and Boll stage 12h after irrigation in 15-cm planting spacing. **(C)** Flowering and Boll stage 3d after irrigation in 30-cm planting spacing. **(D)** Flowering and boll stage 3d after irrigation in 15-cm planting spacing.

The roots of the cotton group grew toward areas of high soil moisture, which influenced the development of their root system architecture. The distribution of soil matric suction played a crucial role in this process. When the same irrigation quota was used, the soil water suction distribution in the soil wetting zone of both 30- and 15-cm planting spacing showed that the further away from the irrigation point, the greater the soil water suction. However, since the planting spacing in 30 cm was twice as larger as that in 15-cm planting, the soil moisture environment for the cotton group roots in 15-cm planting spacing was found to be better than that in 30-cm planting spacing, as shown in **Figure 4**.

**Figure 5:**
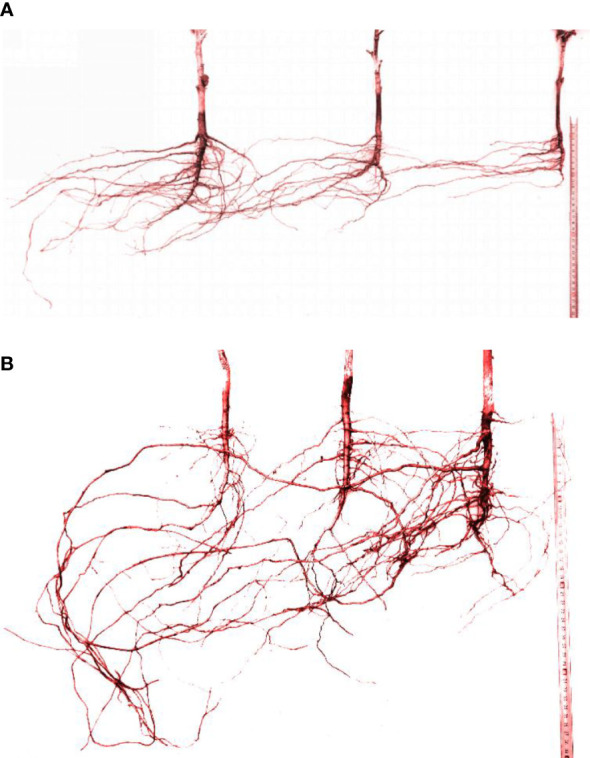
Root system architecture of the cotton group in Boll stage. **(A)** 30-cm planting spacing. **(B)** 15-cm planting spacing.

After 12 hours of irrigation for 30-cm planting spacing (including two cotton plants, **Figures 1A**, 4A), the water stress degree was small. However, the soil water suction at the depth of 0- to 20-cm soil layer was less than 1.5 MPa at the horizontal distance of 70–100 cm from the irrigation point (the position of cotton 3). After 3 days of irrigation (**Figure 4C**), only the soil water suction at the 15- to 20-cm soil depth within 40 cm from the horizontal distance of the irrigation point (including cotton 1) was less than 1.5 MPa in 30-cm planting spacing, the cotton in other locations was severely water stressed. It indicated that cotton was most susceptible to water stress under this treatment. In particular, cotton 3 was always under water stress conditions, so that the root system of cotton 3 not only grew hydrotropism but also had few lateral roots (**Figure 5A**). After 12h of irrigation for 15-cm planting spacing (**Figure 4B**), the soil water suction at the depth of 0–60 cm was far less than 1.5 MPa within 50 cm of the horizontal distance from the irrigation point (including three cotton plants, **Figure 1B**). After 3 days of irrigation (**Figure 4D**), the cotton was subjected to water stress of the lower soil layer in 15-cm planting spacing, only the soil water suction of 0- to 30-cm soil layer was less than 1.5 MPa. The soil water suction of the soil layer below 0- to 30-cm was all greater than 1.5 MPa. Among them, cotton 3 received relatively greater water stress. The results showed that the third cotton plant with 15-cm planting spacing was affected by intermittent water stress. Despite this, the root system exhibited hydrotropism, and the lateral root density was found to be higher than that observed in plant grown with 30-cm planting spacing.

**Figure 6:**
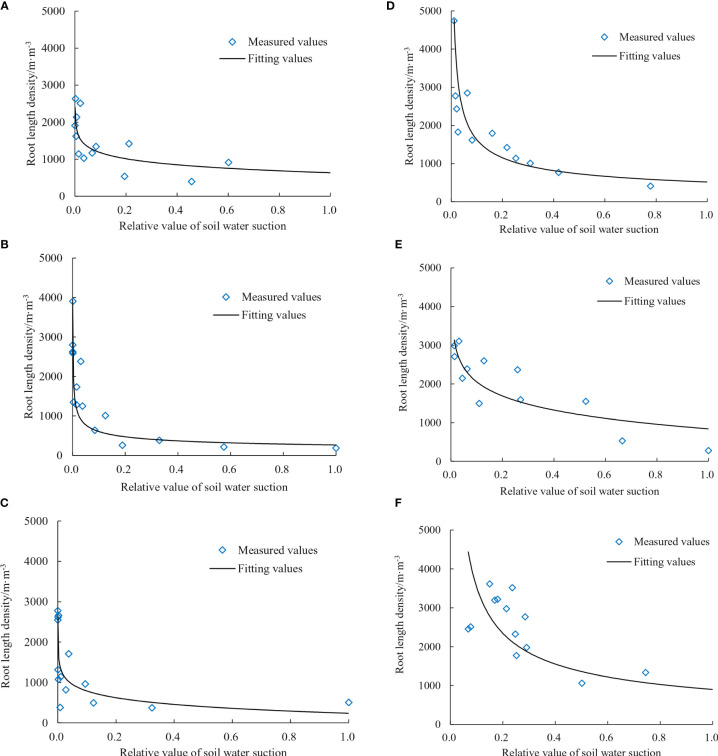
Relationship between root length density of the cotton group and soil water suction. **(A)** Bud stage of 30-cm planting spacing. **(B)** Bud stage of 15-cm planting spacing. **(C)** Flowering stage of 30-cm planting spacing. **(D)** Flowering stage of 15-cm planting spacing. **(E)** Boll stage of 30-cm planting spacing. **(F)** Boll stage of 15-cm planting spacing.

To account for variations in the soil water characteristic curves across different parts of the leaching-pond, the soil water suction of each leaching-pond was expressed as relative values. This enabled the establishment of a unified dynamic relationship for root architecture development. The relative values of soil water suction were normalized using Min-Max Normalization. Additionally, curve fitting was performed after averaging the data, as shown in **Figure 6**. Since the planting spacing of cotton group at 30 cm is wider, a single cotton plant’s root system can occupy a greater volume of soil space, but its root length density is lower. As a result, the reduction in root density with an increase in soil water potential is relatively small for the cotton plants planted at 30 cm. When cotton plants are spaced 15 cm apart during planting, the available space for each plant’s root system is relatively small. As a result, the root density increases and the roots overlap more within a limited soil moisture zone; the root length density decreases more rapidly with soil water suction compared with 30 cm.

### 3.2 Relationship between root density and soil water suction

**Figure 7:**
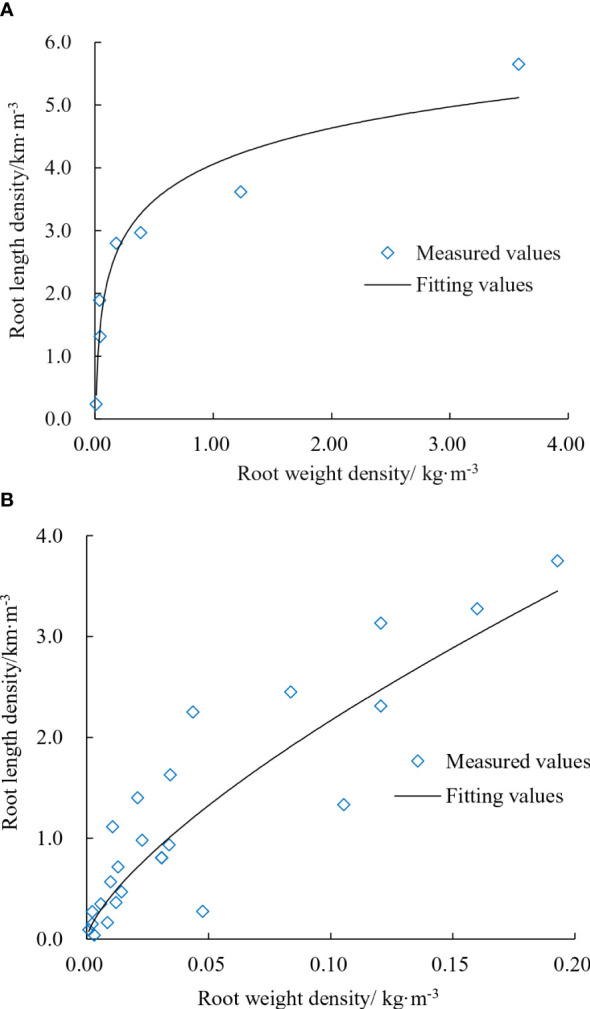
Fitting relationship of root density. **(A)** Root axis location. **(B)** Other locations.

In this study, root density includes root length density and root weight density. The fitting relationship of the root axis location and other locations is shown in **Figure 7**. As the plant location in the leaching-pond is stationary, the main root (root axis location) thickened as the root growth, while the length of lateral roots (other locations) mostly increased. For the main root, root weight and thickness increased with root diameter (**Figure 7A**). For lateral roots, the relationship between root weight density and root length density was nearly linear (**Figure 7B**). The findings indicated that the lateral root diameter was mostly fixed, and root weight increased linearly with root length. Analysis of the dispersion between main and lateral roots based on the fitted relationship of root density reveals that when the root weight density reaches a certain level, the root length density no longer increases. At this point, there are few lateral roots near the main root, and the lateral roots grow in the space away from the main root. No new lateral roots emerge from the main root.

The experiment results showed that the growth status of the cotton root system was affected by the degree and methods of water stress, resulting in significant differences in the final root architecture showed. Therefore, there exists a close relationship between the spatial distribution of soil water and the distribution of cotton root architecture. The relationship between root length density and soil water suction differed between the 15- and 30-cm planting spacing due to the plants farther away from the irrigation point being at the edge of the soil wetting zone under water stress. By fitting the curve (Equation 4 corresponds to the 30-cm planting spacing, and Equation 5 corresponds to the 15-cm planting spacing.), the parameter values in the relationship can be obtained, as shown in **Table 3**. The relationship between root length density and soil water suction follows a logarithmic and a power function relationship at planting spacings of 30 and 15 cm, respectively. The range of fitting accuracy for root length density and soil water suction at a planting spacing of 30 cm is 0.755–0.847, while the corresponding accuracy range for a planting spacing of 15 cm is 0.865–0.895.

**Table 3:** Fitting parameters of the fitting curve of root length density and soil water suction.

Fitting parameters	a	b	c	d	*R* ^2^ (30 cm)	*R* ^2^ (15 cm)
Bud stage	-235.436	1973.302	1643.095	-0.014	0.755	0.895
Flowering stage	-239.590	2335.079	2834.931	-0.126	0.766	0.865
Boll stage	-528.167	3373.453	3957.418	-0.253	0.847	0.894

### 3.3 Root architecture of the cotton group

Roots tend to grow toward areas where water suction is lower, indicating higher water availability. This causes the root architecture to deform, and the distribution of lateral roots is no longer symmetrical along the main root. **Figure 5** showed the root system architecture of the cotton group in the Boll stage (August 18) for 30- and 15-cm planting spacing (the left side was the irrigation point, and the right side was cotton 1, cotton 2 is middle cotton, and cotton 3 is the cotton far from the irrigation point, as shown in **Figure 1**. Removing the plant changed less of the root structure, but it does not affect the overall of the root system architecture.).

In 30-cm planting spacing (**Figure 5A**), the average lateral root diameters of cottons 1, 2, and 3 were 2.96, 2.89, 2.06 mm, and the average lateral root lengths were 66.1, 46.7, and 48.0 cm, respectively. The average angle between the main and lateral roots was greater than 80°, among them, the lateral roots of cotton 3 grow toward the irrigation point at a direction close to 90°. The number of lateral roots of cotton 2 growing in the direction of irrigation point accounted for more than 90% of the total number of lateral roots. The cotton 3 far away from the irrigation point all grew in the direction of the irrigation point, but the number of lateral roots was only 30–40% of the number of lateral roots of cotton 1. In 15-cm planting spacing (**Figure 5B**), the average lateral root diameters of cottons 1, 2, and 3 were 1.86, 2.26, 3.38 mm, and the average lateral root lengths were 62.1, 65.6, and 71.2 cm, respectively. The average value of the main root and lateral root angle was about 70°. The lateral root diameter of cotton 2 and 3 became larger, especially the maximum diameter of the lateral root of cotton 3 reached 5.68 mm, and the longest lateral root was 121.6 cm. The number of lateral roots of cottons 1 and 2 was basically the same, while the number of lateral roots of cotton 3 was significantly larger than the two. The results showed that the lateral root length of the cotton root increased and the angle between the main and lateral roots decreased with the decrease in the water stress degree. The roots of the group plants had different tropisms and competitive growing states.

### 3.4 Simulation of root system architecture

**Figure 8:**
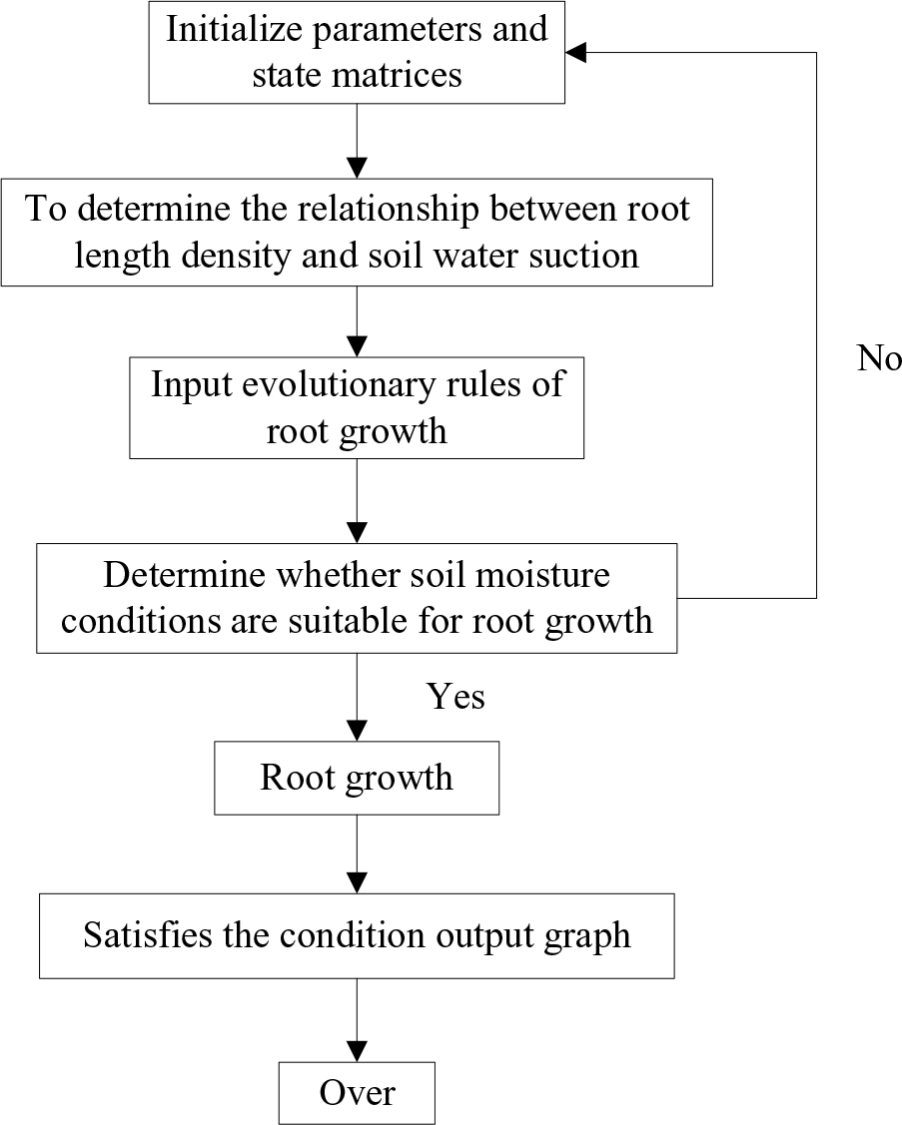
Flow chart of root simulation.

**Figure 9:**
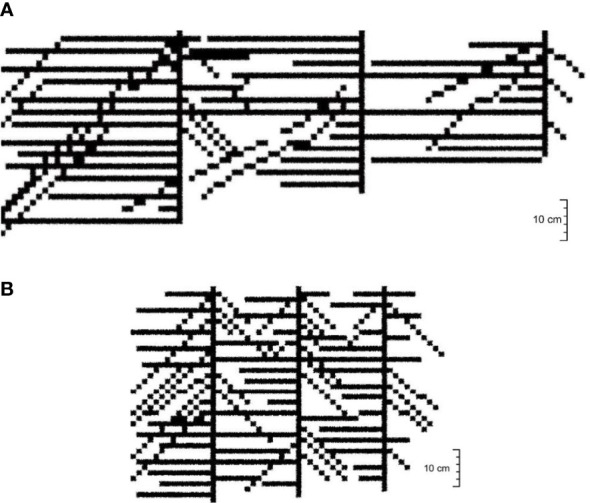
Simulation effect diagram of root system architecture of the cotton group. **(A)** 30-cm Planting spacing. **(B)** 15-cm planting spacing.

The simulation process of root growth under CA model is shown in **Figure 8**. When using the code (Appendix 1) to simulate, the soil volumetric water content (θ*v*) used was the measured value in the experiment, and the soil water content in the CA grid (0.5 cm × 0.5 cm) was subdivided by interpolation. After inputting the Equation (1) (*S*, θ*v*) into the model, the functional relation Equation(4, 5) ( $R_{L}\left(S\right)$ ) was then inputted for cyclic calculation. Initially, the simulated diagram was unclear in terms of the response of the root distribution. However, it clearly reflected the extent of the soil moisture zone, indicating that the root system grows primarily in the moisture zone (**Supplementary Figure S2**). This shows the range of root growth, whereas the essence of root-to-water growth indicates that the soil moisture distribution should be present in the root-growing zones, but not necessarily in soil moisture distribution zones. After formatting the function (*imshow*), the simulation effect diagram of the cotton group in the Boll stage of 30- and 15-cm planting spacing was shown in **Figure 9**.

The root system architecture of the cotton group obtained by the CA model was consistent with the parameters observed in the experiment, such as mostly the angle between the tap root and lateral root was 90° for planting spacing is 30 cm (**Figure 9A**) and not shown for planting spacing is 15 cm (**Figure 9B**). The cotton root architecture of each treatment showed the characteristics of growth hydrotropism (horizontal growth of lateral root). However, the number of lateral root of the “umbrella”-shaped root architecture was less when subjected to strong water stress or longer stress time. Although the number of lateral roots was relatively more under shorter water stress. In the case of a 30-cm planting spacing, the cotton roots extended toward the irrigation point, with the angles between the main root and lateral root being mostly 90°, accounting for about 68.0% of the total number in the experiment. The remaining angles were around 45°–50°, and these results were relatively consistent. While the simulation results for 15-cm planting spacing showed that the lateral root overlapped densely, and the angle between the main and lateral root was large, which was consistent with the observation that the overlap of cotton group root increased with the growth stage.

The root density of the three cotton plants under 30-cm planting spacing differed greatly. The root density of cotton near the irrigation point was always high during the growth stage. The cotton root at the edge of the soil wetting zone grew slowly, and the root density was always small. The root system architecture of the cotton group of 15-cm planting spacing was significantly different in the early stage of the growth. The root density far away from the irrigation point was relatively small. However, the root density of the three cotton plants gradually approached as the growth stage progressed.

## 4 Discussions

### 4.1 Effect of soil water on root growth

In this study, the fertilizer was irrigated with water, and the spatial distribution of fertilizer in the soil was basically the same as that of soil moisture. Therefore, the relationship between the spatial distribution of soil water suction and root length density of cotton group was selected as the driving force for the development of cotton root architecture, to reflect the growing trend of root system architecture of the cotton group. The root system will grow toward the most suitable water and fertilizer environment. The growth of cotton roots depends on the accumulation of root biomass, and its growth process could be expressed by root length density. Under different soil moisture environments, the root length density of cotton group is not the same.

As the surrounding and bottom of the leaching-pond were separated by plastic film, it was difficult for groundwater and surrounding soil water to supply water to the cotton root layer. After 3 days of irrigation (**Figure 4C**), some of the water infiltrated into the soil below the root layer, but it is sucked back to the root layer by the action of subsequent root water absorption, resulting in an increase in the soil matrix suction below the root layer. Because the water seeping from irrigation is in the soil, gravity and water pressure action are involved. The soil water content decreases as the water leaks out, and the suction force of the soil gradually increases, sucking back the water seeping into the soil (Filipović, 2020). Therefore, it is necessary to analyze the correlation between root growth parameters and soil moisture parameters in the modeling process (Guswa, 2010) and root degree of plasticity in the soil environment (Hu et al., 2006).

The relationship between the root weight density and the root length density of the lateral root was almost linear, indicating that the diameter of the lateral root was almost fixed and the weight of the root increased as the root length increased (**Figure 7B**). The relationship between root length density and root weight density lays the foundation for simulating root diameter and helps to improve the accuracy of the model. The larger the root diameter, the heavier the root weight and the thicker the simulated root is. When the degree of dispersion between the main and lateral roots was examined using the fitting relationship of root density, it was discovered that the root weight density increased to a certain extent while the root length density did not. There were few lateral roots near the main root at this time, and the lateral roots extended to the space away from the main root. A new lateral root has not been formed on the main root. In addition to soil environmental factors, ecological factors of the cotton root have an impact on the simulation accuracy of the root system architecture. The relationship between the significant changes in root diameter throughout the whole growth stages of cotton and the driving forces selected in the model needs to be further investigated.

The decrease in root length density with increasing soil water suction reflects the effect of matric potential on root growth, as predicted by physical models to a greater extent (de Jong van Lier et al., 2008; Shimazaki et al., 2015; Ng et al., 2016). However, we found that the competition for soil water between cotton plants has intensified, resulting in a reduced in total root volume and average root length. The degree of root overlap was intensified in areas with limited soil moisture, resulting in a significant reduction in the water absorption capacity of the root system. When soil water suction increased, root elongation rate decreased (Leung et al., 2015). Plant physiological and ecological mechanisms showed that root distribution and plant growth interact are proportional to plant size and root characteristics (McPhee and Aarssen, 2001). In this study, the root length density of the cotton group decreased with increasing soil water suction, which was consistent with the physiological and ecological mechanism of root growth, that is, the roots grew toward water (**Figure 3**). The growth of root density was closely related to root suction at different planting spacing. The results showed that the cotton root length density decreased slightly as soil water suction increased in 30-cm planting spacing. Cotton planting spacing in 15 cm was small when compared with 30-cm planting spacing. The space occupied by only one cotton root system was smaller, so the root was denser and the root density was high. The simulated results were found to be in good agreement with the experimental observation, highlighting the effectiveness of the proposed research methodology and exhibiting hydrotropism and competitive growth, which need to further investigation. Additionally, the growth characteristics of the cotton plants were reflected in the simulated results (Supplementary Plant analysis, **Supplementary Figure S3**). It was found that altered root growth affected the cotton canopy, with weak growth observed in plants with a planting spacing of 30 cm, while vigorous growth was observed in plants with a spacing of 15 cm.

### 4.2 Analysis of the accuracy of CA model

The CA method, used to establish the root growth model of cotton group, is based on the fractal theory. Theoretical knowledge includes the self-similar development of the root system according to a certain law, the hydrotropism of root growth. The fractal dimension reflects the basic characteristics and spatial distribution of root water uptake and serves as an indication of root water uptake capacity. This method is compatible with the root growth of the cotton group within the range of the soil wetting area, as both rely on soil moisture as a factor in guiding root growth through hydrotropism. The results of this study reflect the self-similarity characteristics of root extension pathways in the soil.

**Figure 10:**
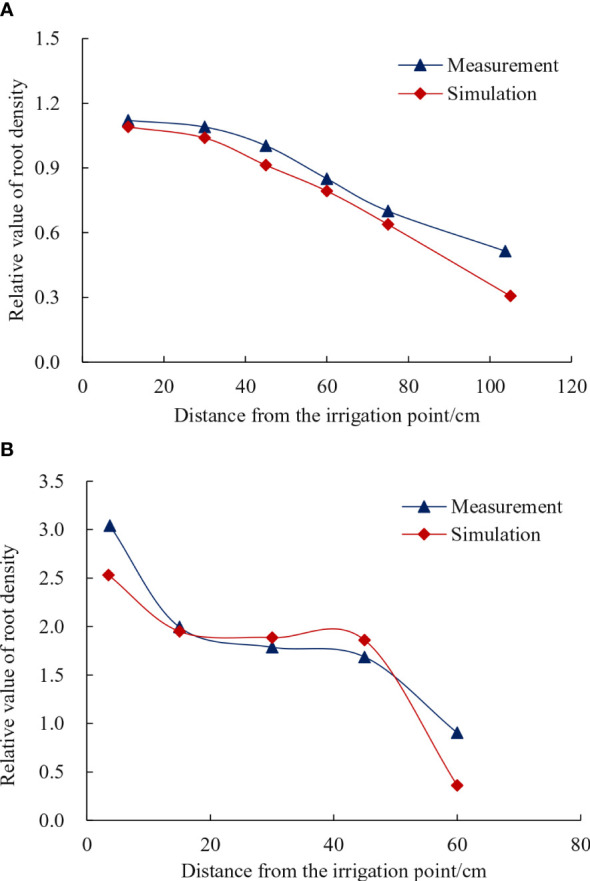
Comparison of relative values of root length density. **(A)** 30-cm planting spacing. **(B)** 15-cm planting spacing.

The root density was simulated using CA model and plotted base on the grid density of root occupation. The resulting root coverage rate exhibited a trend consistent with the distribution of relative value of cotton root density observed in the experiment, as shown in **Figure 10**. The comparison between the experimental results for 30- and 15-cm planting spacing and the trend line of relative root density values simulated by the model revealed simulation errors of 6.03 and 15.04%, respectively. The CA was thus utilized to simulate the distribution of the cotton group’s root system architecture, relying on the relationship function between root length density and soil water suction. This approach effectively reflects the key characteristics of root growth, which involves the uptake of water by the roots.

The error generated when using CA to simulate the root structure of cotton group was not only related to the uneven distribution of soil moisture and root physiological factors but also related to the soil pore structure and its changes. Soil was considered as a homogeneous porous medium in the modeling, but in fact many large pores would appear in the soil under alternate dry and wet conditions. In fact, there will be many large pores in the soil under the alternating action of dry and wet conditions (Deepagoda et al., 2011; Amoakwaha et al., 2017), and the distribution of macropores often has statistical fractal characteristics (Martínez et al., 2010; Almquista et al., 2018; Mohammadi et al., 2019). The root tips begin to bend after sensing the moisture and nutrients in the soil, forming the preferred path for plant root growth (Leung et al., 2015; Shimazaki et al., 2015). If the physical characteristics of the pores (pore size, curvature, etc.) were considered in the root architecture model, the process of root growth with bending might be reproduced and the simulation accuracy can be improved. It was showed in this research, the smaller the CA grid is, the more accurate the model will be, and the closer the simulated root architecture will be to the actual root shape.

The CA model has a wide range of applications, including simulating root growth through grid-based modeling, predicting the distribution of plant growth and soil moisture, and providing support for water resource management and environmental protection. This method can also be used to study the effects of different pollutants on plant roots, predict the distribution and growth of roots in polluted soil, and evaluate the root morphology and growth characteristics of different crop varieties, providing a scientific basis for crop variety screening and improvement. In summary, the CA model is an effective tool for researchers to understand and manage natural systems such as plants, soils, and ecosystems.

## 5 Conclusions

In this study, we used the CA model to simulate the root architecture of cotton group base on the function of root length density and soil water suction. The relationship between root length density and soil water suction followed logarithmic and power functions for the cotton plants with a planting spacing of 30 and 15 cm, respectively. This similarity of the root architecture simulation was determined by the fineness of the 2D grid division in the soil wetting area. The simulation results indicated a significant agreement between the visualized root architecture of each treatment and the experimental observations. Notably, the lateral roots showed horizontal growth at 30-cm planting spacing of cotton group, whereas at 15-cm spacing, they were densely overlapped. The simulation errors for the 30- and 15-cm planting spacing were 6.03 and 15.04%, respectively. In addition, when planted with a spacing of 30 cm, cotton plants were found to be sensitive to water stress due to their relatively shallow root system. The lateral roots of the cotton plants grew almost 90° away from the irrigation point. In contrast, when planted with a spacing of 15 cm, only the cotton plants located far from the irrigation point experienced intermittent water stress. In this case, the lateral roots of the cotton plants were thick, overlapping, and competed for resources. Our simulations have yielded insights into the root system architecture of cotton plants grown under varying planting spacing and water stress conditions. These results serve as a foundational basis for developing theories on crop planting spacing for future applications.

## Data availability statement

The original contributions presented in the study are included in the article/**Supplementary Material**. Further inquiries can be directed to the corresponding authors.

## Author contributions

CG: Data curation, Writing- Original draft preparation. ML: Supervision, Project administration. DL: Writing- Reviewing and Editing. All authors contributed to the article and approved the submitted version.
