# Supplementary material for: Root architecture and visualization model of cotton group with different planting spacing under local irrigation
Source: Front Plant Sci. 2023 Apr 21;14:1080234. doi: 10.3389/fpls.2023.1080234 (PMC10160472; doi:10.3389/fpls.2023.1080234)
Supplement: Supplementary file 1 [file DataSheet_1.docx]

**Captions of Supplementary figures**

**Figure S1**. Actual picture of leaching-pond.

**Figure S2**. The initially simulated diagram of the root distribution.

**Figure S3.** Plant changes with the days after seeding. (a) Average plant height. (b) Total leaf area.


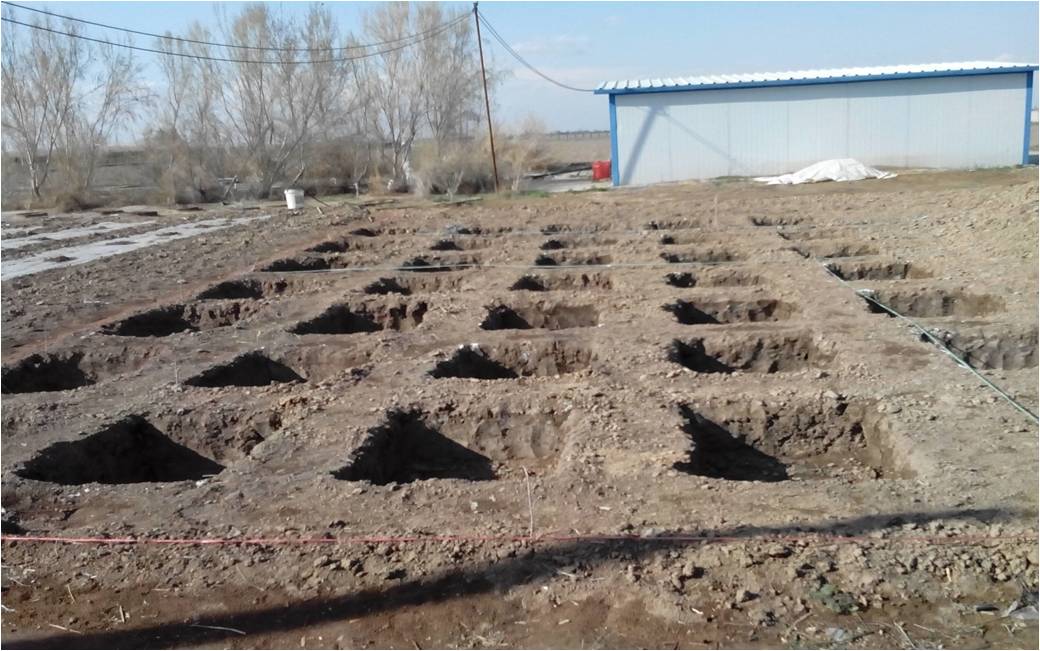


**Figure S1**. Actual picture of leaching-pond.

**
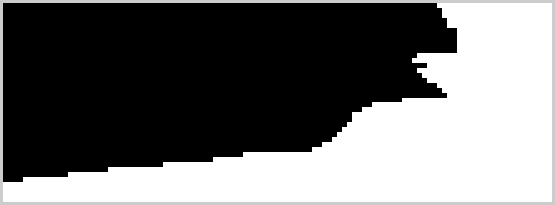
**

**Figure S2**. The initially simulated diagram of the root distribution.

(a)

(b)

**Figure S3.** Plant changes with the days after seeding. (a) Average plant height. (b) Total leaf area.
