# Supplementary material for: Root architecture and visualization model of cotton group with different planting spacing under local irrigation
Source: Front Plant Sci. 2023 Apr 21;14:1080234. doi: 10.3389/fpls.2023.1080234 (PMC10160472; doi:10.3389/fpls.2023.1080234)
Supplement: Supplementary file 2 [file DataSheet_2.docx]

## CA program code

Baesd on the specific steps given in the flowchart, the initial status was defined as 0. The status matrix was determined by root density as well as soil moisture distribution. and *MATLAB* has been used to write the *CA* model based on *imshow* function for simulation. The main code of the algorithm instruction program code was as follows:

%--------------Read data-------------------------------------------------

*S = 122977912.013 * exp(-0.729*water);*

*RLD=-235.437 * log(S) + 1973.302;*

*root_S = zeros (40,110);*

*root_S = root_S + RLD;*

*root_desity = ones (40,110);*

*for i =1: size(root_S,1)*

*for j =1: size(root_S,2)*

*if root_S(i,j) > 0*

*root_desity (i,j) = 0;*

*end*

*end*

*end*

*%--------------Output----------------*

*figure*

*imshow (root_desity, root range) % 0 means black, 1 means white*

*The format of the function call was：*

*Imshow (l, n);*
